# “CULTIVA MENTE”: DESIGNING A BRAIN HEALTH CAMPAIGN FOR PROMOTING DEMENTIA-RISK REDUCTION

**DOI:** 10.1093/geroni/igae098.1933

**Published:** 2024-12-31

**Authors:** Jose Aravena, Hugo Castro, Ronald Poblete, Becca Levy

**Affiliations:** Department of Social & Behavioral Sciences, Yale University, New Haven, Connecticut, United States; Inmedios, Santiago, Region Metropolitana, Chile; Unaffiliated, Santiago, Region Metropolitana, Chile; Yale University School of Public Health, New Haven, Connecticut, United States

## Abstract

Implementing a successful campaign to promote dementia-risk reduction requires an understanding of how both older persons and healthcare providers conceptualize dementia risk as well as culturally relevant barriers and facilitating factors related to achieving brain health. Thus, this study aimed to achieve this goal through a qualitative study using a design-thinking approach with senior center participants aged 60 and older with no dementia, and healthcare professionals working at the senior centers in Chile. Ten centers, each with one focus group of senior center participants and one focus group of health professionals discussed topics related to a brain health campaign. Thematic analysis was conducted and supplemented with an analysis of previous educational campaigns. A total of 115 patients and 51 health professionals participated. Themes identified included: A) social images about dementia; B) knowledge about dementia risk factors; C) structural barriers to dementia prevention, including ageism; D) barriers and facilitating factors to dementia prevention, such as education: and E) role models of persons that worked toward brain health. Based on these results and analysis of previous initiatives, an educational campaign named ‘Cultiva mente’ (cultivate mind) was developed, referring metaphorically to nurturing minds like plants. The campaign includes posters and brochures highlighting positive images and behaviors about aging and brain health, an educational interactive website, and a training program for health professionals on dementia risk reduction strategies. This study constitutes the design of a culturally relevant campaign to promote dementia risk reduction that will be tested in a future efficacy study

